# Primary surgery to prevent hip dislocation in children with cerebral palsy in Sweden: a minimum 5-year follow-up by the national surveillance program (CPUP)

**DOI:** 10.1080/17453674.2019.1627116

**Published:** 2019-06-18

**Authors:** Nikolaos Kiapekos, Eva Broström, Gunnar Hägglund, Per Åstrand

**Affiliations:** a Department of Women’s and Children’s Health, Karolinska Institute, Karolinska University Hospital, Stockholm;; b Department of Pediatric Orthopedics, Astrid Lindgren’s Children Hospital, Karolinska University Hospital, Stockholm;; c Lund University, Department of Clinical Sciences, Lund, Orthopedics, Lund, Sweden

## Abstract

Background and purpose — Children with cerebral palsy (CP) have an increased risk of hip dislocation. Outcome studies after surgery to prevent hip dislocation in children with CP are usually retrospective series from single tertiary referral centers. According to the national CP surveillance program in Sweden (CPUP), hip surgery should preferably be performed at an early age to prevent hip dislocation. Preventive operations are performed in 12 different Swedish hospitals. We compared the outcomes between soft tissue release and femoral osteotomy in children with CP treated in these hospitals.

Patients and methods — 186 children with CP underwent either adductor–iliopsoas tenotomy (APT) or femoral osteotomy (FO) as the primary, preventive surgery because of hip displacement. They were followed for a minimum of 5 years (mean 8 years) regarding revision surgery and hip migration. A good outcome was defined as the absence of revision surgery and a migration percentage (MP) < 50% at the latest follow-up. Logistic and Cox regression analysis were used to investigate the influence of age, sex, preoperative MP, Gross Motor Function Classification System (GMFCS) level, and CP subtype.

Results — APT was performed in 129 (69%) children. After 5 years, the reoperation rate was 43%, and 2 children (2%) had an MP > 50%. For the 57 children who underwent FO, the corresponding figures were 39% and 9%. Of the potential risk factors studied, the outcome was statistically significantly associated with preoperative MP only in children who underwent APT, but not in those who underwent FO. None of the other factors were significantly associated with the outcome in the 2 procedure groups.

Interpretation — Reoperation rates after preventive surgery are high and indicate the importance of continued postoperative follow-up. Age, sex, GMFCS level, and CP subtype did not influence the outcome significantly.

In children with cerebral palsy (CP), the risk of hip displacement increases with decreasing gross motor function and may approach 90% in the most-affected children (Morton et al. 2006, Soo et al. 2006, Hägglund et al. 2007). Surveillance programs can be effective in identifying children with an increased risk of hip dislocation (Gordon and Simkiss 2006, Hägglund et al. 2014), but there are no current recommendations for the timing of a specific type of surgical procedure (Stott and Piedrahita 2004, Bouwhuis et al. 2015).

In Sweden, the national surveillance program for children with CP (CPUP) includes almost all children (> 95%) with CP born in 2002 or later. In the CPUP, children with CP undergo regular radiographic examinations to detect early hip displacement, and preventive surgery is recommended when the migration percentage (MP) exceeds 40%. However, the CPUP makes no specific recommendations about when adductor–iliopsoas tenotomy (APT) or femoral osteotomy (FO) should be performed.

Previous studies have shown that soft tissue release (e.g., APT) is more effective in children capable of walking and when the MP is < 50% (Turker and Lee 2000, Presedo et al. 2005, Shore et al. 2012, Terjesen 2017). Soft tissue release is commonly used in younger children and its use implies shorter operation time with fewer complications as well as a shorter and easier postoperative rehabilitation. Osseous procedures are usually used in older children and in children with more severe displacement (MP > 50%), but they are associated with longer rehabilitation and more complications (Bouwhuis et al. 2015).

Generally, outcome studies after hip surgery in children with CP have been reported by tertiary referral centers. A recent report noted that the surgeon’s experience may have a major influence on the outcome after FO (Shore et al. 2015). In Sweden, surgical procedures to prevent hip dislocation in children with CP are performed in 7 university hospitals and 5 regional hospitals, all of which were included in this study.

The aim of this population-based study was to compare the outcomes between 2 different surgical procedures (APT or FO) used for prevention of hip dislocation. Outcome was assessed by reoperation rates and extent of hip migration. Logistic as well as Cox regression analysis was used to investigate the influence of age at the time of surgery, sex, preoperative MP, Gross Motor Function Classification System (GMFCS) level, and CP subtype.

## Patients and methods

This was a population-based register study of children with CP who were followed in the Swedish national quality register CPUP. It is estimated that the register includes more than 95% of the children with CP in Sweden (Westbom et al. 2007). At the end of the treatment period in this study (2012), 2,948 children up to 18 years of age were registered in the CPUP, and 1,238 of them were classified as GMFCS levels III–V. At that time, 13 children (8 girls) had an MP = 100% but were considered too weak for surgery (CPUP annual report 2013, ISBN 978-91-980722-0-4).

The CPUP includes continuous standardized follow-up of gross and fine motor function, clinical findings and treatment. The CP diagnosis and subtype are reported by the child’s neuropediatrician. The dominant symptom is also classified by the child’s physiotherapist as spastic, dyskinetic, or mixed. 4 children classified with mixed symptoms and 1 child with ataxia were omitted from the statistical analyses based on CP subtype. The physiotherapist also classifies each child’s gross motor function according to the GMFCS (Palisano et al. 1997).

A local coordinator is responsible for reporting the orthopedic surgical procedures to CPUP, using a recording form specifying the different types of procedures. The types of pre- and postoperative treatment were not investigated in this study.

Anteroposterior radiographs of the hips in the supine position are acquired at the child’s inclusion in the register and then at least once a year until 8 years of age in children with GMFCS III–V. After that, the follow-up is continued on an individual basis. The MP is calculated according to Reimers’ index (1980).

This study included all children in the CPUP register operated on primarily for hip displacement with either bilateral APT or uni- or bilateral FO with or without concurrent pelvic osteotomy (PO), with a minimum of 5 years of follow-up, i.e., children treated during the period 1995–2012.

The criteria for failure were either a second hip operation (on any side) or MP > 50% at the latest radiological follow-up. The treatment of failure was further divided into 3 categories: (1) reoperation using soft tissue hip procedures (APT or adductor tenotomies), (2) reoperation using bony procedures (FO with or without PO), and (3) no reoperation but an MP > 50% in at least 1 hip. For children who were operated on with unilateral FO the side of the reoperation (ipsilateral or contralateral) was also specified. Removal of osteosynthesis material was not considered as revision surgery. In the statistical analysis, the influence of surgical volume on the outcome after FO was studied by identifying hospitals with a “high surgical volume unit” which was defined as the 3 larger university clinics that treated 37 of the 57 children who underwent FO.

The inclusion criteria were: (1) confirmed CP diagnosis, (2) bilateral APT or uni- or bilateral FO as the index surgery, (3) MP >30% in at least 1 hip preoperatively before the first operation, and (4) a minimum 5 years of follow-up after the index surgery.

Children undergoing unilateral (2 children) or bilateral adductor surgery without iliopsoas lengthening or tenotomy (7 children) were thus excluded. The rationale for including only children who underwent bilateral APT was to diminish variations in the surgical “dosage” (e.g., percutaneous adductor tenotomies).

A total of 192 children fulfilled the inclusion criteria. 2 children moved abroad and 4 children died before the 5-year follow-up, and were therefore excluded. The remaining 186 children (79 girls) were followed for a minimum of 5 years and had a mean follow-up time of 8 (5–20) years.

### Statistics

Continuous data are presented as mean (SD), and categorical data are presented as frequency count and percentage. When comparing the groups of children undergoing APT or FO, continuous data were analyzed using Student’s t-test or the Mann–Whitney U-test. The chi-square test was used to analyze categorical data. A p-value < 0.05 was considered to be significant. IBM SPSS Statistics (version 23; IBM Corp, Armonk, NY, USA) was used for these analyses. Logistic regression analysis models were constructed with failure as the outcome and age at the time of surgery, sex, preoperative MP, GMFCS level, and CP subtype as covariates. Preoperative MP was scaled in 5% increments. Hazard ratios using Cox regression analysis were also calculated using the same predictors. In 1 logistic regression model of the entire material, the type of surgery (APT vs. FO) was included as a covariate. In another logistic regression model to study the outcome after FO, the covariate “high surgical volume unit” was also included. These analyses were performed using STATA 13 software (Stata Corp, College Station, TX, USA). Kaplan–Meier curves are presented to show the cumulative proportion of failures over the follow-up period.

### Ethics, funding, and conflicts of interest

Ethical approval was obtained from the Regional Ethical Review Board in Lund, Sweden (LU-443-99). The support of Stiftelsen Promobilia, Norrbacka-Eugeniastiftelsen, Josef och Linnéa Carlssons stiftelse, Stiftelsen för bistånd åt rörelsehindrade i Skåne, and Riksförbundet för Rörelsehindrade Barn och Ungdomar is gratefully acknowledged. The authors declare no conflicts of interest.

## Results

Of the 186 children, 129 (69%) underwent bilateral APT and 57 (31%) FO (34 unilateral, 23 bilateral, with or without simultaneous PO) as their primary procedure. Almost 90% of the children operated on were in GMFCS level IV or V (Table 1). To allow for a meaningful statistical analysis, the small numbers of children in GMFCS levels I–III were pooled.

**Table 1.** Characteristics of the children operated on with adductor–iliopsoas tenotomy (APT) or femoral osteotomy (FO). Values are frequency (%) unless otherwise specified

	APT	FO	
Factor	n = 129	n = 57	p-value
Mean age (SD)			
at primary operation	4.9 (2.2)	5.6 (2.3)	0.03
at second operation	6.8 (2.6)	8.7 (2.5)	0.003
Girls	56 (43)	23 (40)	0.8
CP subtype		0.4	
spastic		79 (61)	38 (67)
dyskinetic		47 (36)	17 (30)
GMFCS		0.3	
I + II + III	13 (10)	7 (12)	
IV	37 (29)	10 (18)	
V	79 (61)	40 (70)	
Mean preoperative MP (SD)^a^	47 (12)	58 (16)	0.001
Reoperations at 5 years			
total	56 (43)	22 (39)	0.5
soft tissue reoperations	1 (1)	5 (9)	0.005
bony reoperations	55 (43)	17 (30)	0.1
MP > 50% at 5 years	2 (2)	5 (9)	0.2
Failures at 5 years	58 (45)	27 (47)	0.8
Failures at 5–20 years	62 (48)	30 (53)	0.6

a MP worst hip

GMFCS = Gross Motor Function Classification System level

MP = migration percentage

The children who were operated on with APT were younger than those who were operated on with FO as the index operation (p = 0.03) as well as at the second surgery, and they had a lower mean preoperative MP in the worst hip. 6 (3%) children had APT as the second surgery. There were more soft tissue reoperations after FO than after APT, but GMFCS distribution, sex, CP subtype, other reoperation rates, and failure rates did not differ significantly between groups (Table 1).

A logistic regression model was created in which age, sex, CP subtype, GMFCS level, and MP were adjusted, and the type of operation (APT or FO) was included as a covariate. APT was selected as the reference. No statistically significant association was found between the type of operation and failure (odds ratio = 0.6, 95% CI = 0.3–1.5). The only statistically significant predictor found was the preoperative MP (odds ratio = 1.3, 95% CI = 1.1–1.6).

Of the 129 children who were operated on with an APT, 56 (43%) were reoperated on. All of these 56 children were operated on with FO (in combination with PO in 15 children) within 5 years, and 2 children (2%) had an MP > 50% at the 5-year follow-up. In 1 child (1%), a repeat APT was performed 6 months before the FO. The other 55 children (43%) were reoperated on with FO with or without simultaneous PO. The revision surgery was performed on average 2.3 years (0.5–7) after the index operation. 1 child received a reoperation after more than 5 years. The highest preoperative MP that improved after APT and was not associated with the need for a reoperation was 56%.

In the logistic regression analysis, only preoperative MP was statistically significant and the other factors studied (age at the time of surgery, sex, GMFCS level, and CP subtype) were not significant predictors of failure (Table 2). In the Cox regression analysis of the hazard ratios, only preoperative MP was a statistically significant predictor of failure (Table 2). In a separate logistic regression model in which children with GMFCS IV and V were pooled, the preoperative MP was the only statistically significant predictor for failure (data not shown).

**Table 2.** Statistical analyses of potential risk factors for failure after adductor–psoas tenotomy

	Odds ratio	Hazard ratio
Variable	(95% CI)	(95% CI)
Age at surgery	0.9 (0.8–1.1)	0.9 (0.8–1.0)
Boy	Reference	Reference
Girl	0.6 (0.3–1.4)	0.7 (0.4–1.1)
CP subtype, spastic	Reference	Reference
dyskinetic	1.5 (0.6–3.5)	1.1 (0.6–1.9)
GMFCS I + II + III	Reference	Reference
IV	2.1 (0.4–10.4)	1.7 (0.5–6.1)
V	2.7 (0.6–12.4)	2.1 (0.6–7)
MP, 5% increase	1.5 (1.1–1.8)	1.2 (1.1–1.3)

For abbreviations, see Table 1.

**Figure 1.**
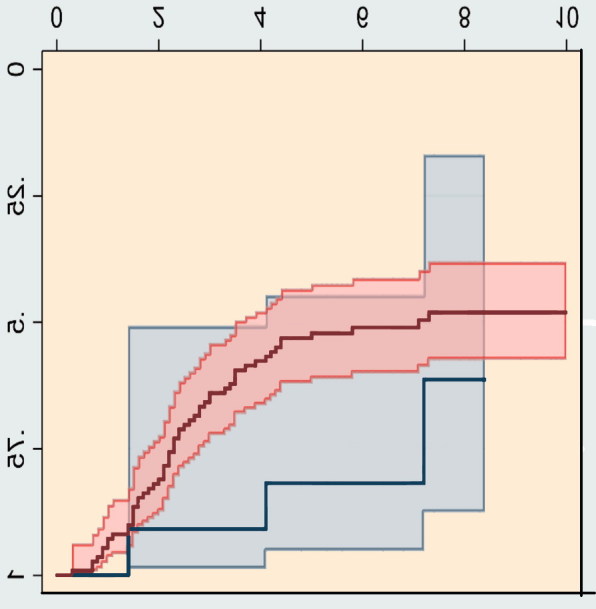
Kaplan–Meier curves showing the proportion of failures with time after the index operation, bilateral adductor–psoas tenotomy, grouped according to the Gross Motor Function Classification System (GMFCS). GMFCS I–III and GMFCS IV–V are pooled for comparison of children who rely on a wheelchair for transport (GMFCS IV–V) with children more capable of walking (GMFCS I–III). Shaded areas represent 95% confidence intervals.

Kaplan–Meier survivorship plots showed a higher failure rate for children in GMFCS IV–V as compared with those with GMFCS I–III (Figure 1), although the difference was not statistically significant. For children treated with APT, the outcomes of 50% of the children in GMFCS V were classified as a failure before the children were 5 years of age (Figure 1).

Of the 57 children who had an FO (in 80 hips) as their primary surgery, 22 children (24 hips, 30%) were reoperated on within 5 years and 2 children after 5 years. The distributions of soft tissue and bony reoperations are given in Table 1. Revision surgery was performed on average 3.6 years (0.8–7) after the index surgery. At the 5-year follow-up, 5 children (9%) had an MP > 50% (Table 1), 23 children (40%) were operated on with a bilateral FO on the first occasion, and 15 children (26%) were operated on with an FO combined with PO (Table 3). The outcome did not differ significantly between groups.

**Table 3.** Distribution of the 57 children operated on with femoral osteotomy

Factor	n	Reoperations	MP > 50%	p-value
Unilateral surgery	34	17	3	
Bilateral surgery	23	7	2	0.6
Femoral osteotomy (FO)	42	17	3	
FO + pelvic osteotomy	15	7	2	0.8

MP = migration percentage

Of the 24 children who received reoperations after FO without PO as primary surgery, 9 were operated on with FO only, 9 with FO and PO, 1 with PO only, and 5 with APT as the second surgery. 9 of the 34 children with unilateral FO as their index operation had an ipsilateral reoperation, and 3 children had revision surgery on the contralateral side. 1 child developed contralateral MP > 50%. The latter 4 children were not classified as failures, when the failure rate was calculated per hip and not per patient.

Of the 15 children who had combined FO and PO, 4 were operated on with an APT as their reoperation.

Similar to the analysis of the outcome of APT, both logistic and Cox regression analyses were performed for the outcome of FO. In this model, we also included as a covariate “high surgical volume unit,” which represented the 3 university clinics that contributed 37 of the 57 patients who had FO as the index surgery. The confidence intervals are large because of the small sample size (Table 4).

**Table 4.** Statistical analyses of potential risk factors for failure after femoral osteotomy

	Odds ratio	Hazard ratio
Variable	(95% CI)	(95% CI)
Age at surgery	0.6 (0.4–1.0)	1 (0.8–1.3)
Boys	Reference	Reference
Girls	0.1 (0.02–1.0)	0.3 (0.1–1.0)
CP subtype, spastic	Reference	Reference
dyskinetic	10.3 (1.1–92)	2.6 (0.9–7.6)
GMFCS I + II + III	Reference	Reference
IV–V	8.0 (0.8–79)	2.6 (0.5–14)
MP, 5% increase	1.3 (0.9–1.7)	1.1 (0.9–1.3)
High surgical volume unit	0.1 (0.01–0.7)	0.6 (0.2–2.1)

For abbreviations, see Table 1.

**Figure 2.**
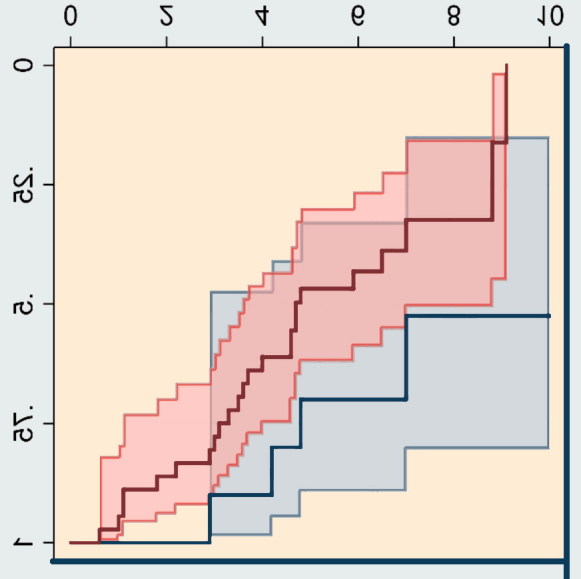
Kaplan–Meier curves showing the proportion of failures with time after the index operation, femoral osteotomy, for all patients. Gross Motor Function Classification System (GMFCS) levels I–III are not shown because of the small numbers of patients. Shaded areas represent 95% confidence intervals. The vertical end of the red curve indicates that the last patient followed was reoperated.

The Kaplan–Meier plot of the outcome for children undergoing FO showed a tendency for increasing differences in reoperation rates between children with GMFCS levels IV or V with increasing follow-up time, but the number of patients followed for more than 5 years was very low (Figure 2).

## Discussion

Our study is based on data from the CPUP Swedish national quality register, which includes > 95% of all children with CP (Westbom et al. 2007). To our knowledge, this is the first national-level, population-based study describing the outcomes after primary hip surgery (APT or FO) to prevent hip dislocation in children with CP. We found similar failure rates for both methods (45% and 47%, respectively, Table 1) after 5 years of follow-up.

Generally, the outcome after surgery for hip displacement in children with CP is influenced by patient selection, duration of follow-up, type(s) of procedure, and varying definitions of success and failure. A shorter follow-up is usually associated with higher success rates. Comparing outcome after FO is complicated by the facts that the reoperation or success rate is often reported per hip and not per patient, and the percentages of hips/patients who undergo PO and FO at the same time vary between studies. It seems likely that the percentage of patients who undergo bilateral FO may also influence the success rate per hip. Choosing contralateral surgery more often for reasons of symmetry in seating and standing rather than increased hip migration may increase the success rate per hip.

APT was performed as the primary procedure in 69% of the children, and as the second procedure in 3%. To facilitate comparisons, we followed the method of Shore et al. (2012), who defined failure as either a reoperation or an MP > 50%. Compared with other studies on children in GMFCS IV–V with sufficiently long follow-up, our failure rate of 48% is higher than the approximately 40% reported by Presedo et al. (2005) and Terjesen (2017), but lower than the 58% reported by Turker and Lee (2000) and 75% by Shore et al. (2012). In our study, the only statistically significant outcome predictor after APT was the preoperative MP, which is consistent with several previous publications (Onimus et al. 1991, Miller et al. 1997, Turker and Lee 2000, Stott and Piedrahita 2004, Terjesen et al. 2005, Terjesen 2017), but contrasts with the study by Shore et al. (2012), who found that GMFCS level was the strongest predictor. However, in that study, the children in GMFCS III, IV, and V were compared with a larger group of children in GMFCS II, who had a very high success rate after APT (94%).

The single highest preoperative MP that did not result in failure after APT was 56%. The logistic and Cox regression analyses showed that an increase in preoperative MP was significantly associated with an increased risk for failure after APT. Even though we did not try to identify a specific threshold MP value for increased risk of failure, we found no reason to change to the commonly recommended threshold value of MP < 50% for considering APT.

Recent reviews indicate that combined FO and PO procedures may have a lower failure rate than FO only (Bouwhuis et al. 2015, El-Sobky et al. 2018). However, in studies that included FO and PO only, the failure rates varied widely from 2% (Rutz et al. 2015) to 47% (Shea et al. 1997). In studies of FO with > 10 years of follow-up, the failure rates range from 20% (Oh et al. 2007) to 44% (Canavese et al. 2010) of hips. The percentage of hips treated with bilateral FO in the material may influence the failure rate per hip. For example, Oh et al. (2007) reported a 20% reoperation rate in 61 hips in 31 children, whereas Canavese et al. (2010) found a 44% reoperation rate after unilateral FO only. In our study, 80 hips were operated on in 57 children, and the reoperation rate per hip was 30% at 5 years. Using the same criteria for failure per patient as for APT above, Shore et al. (2015) reported a failure rate of 37%, which is lower than the 53% in our study. In their study of 567 hips in 320 patients undergoing FO, the strongest predictors were age, GMFCS level, and surgeon volume. The higher failure rate in our study may be explained by the higher percentage of children in GMFCS IV–V (88% vs. 64%) and the lower mean age (5.6 vs. 8.2 years). Shore et al. speculated that their lower failure rates may reflect the high percentage of children who were operated on with combined FO and PO (50%) in their material, which was 26% in our study.

The analysis of the association between FO and outcome predictors was limited by the small patient sample and large confidence intervals (Table 4). In 1 logistic regression model, we studied the effect of surgeon volume on the outcome by including “high surgical volume unit” as a covariate. Although there was a tendency for lower failure rates in these units, the large confidence intervals do not allow us to draw conclusions.

The literature has few studies similar to ours that compared outcome after soft tissue procedures and skeletal reconstruction to prevent hip dislocation in children with CP. However, in another study of a younger group than ours (mean age at the time surgery of 3.9 years, mean follow-up 5 years), the reoperation rate per patient was 77% after soft tissue release and 74% after FO (Schmale et al. 2006).

We found no statistically significant association between the type of operation (APT or FO) and failure rates after adjusting for age and MP. The failure rates after FO are within the ranges reported in similar studies, and it is possible that these numbers may improve by centralizing FO surgery to only a few hospitals. It seems appropriate to continue to regard APT as a valuable surgical option for young children with CP with early hip displacement. Preferably, the operation should be performed while the MP is < 50%. For APT as well as FO, the parents should be informed that there is a substantial risk of the need for reoperation within the following few years.

In conclusion, surgical prevention of hip dislocation in children with CP is associated with a high reoperation rate, both after soft tissue release and after reconstructive osteotomy. Continued postoperative hip surveillance is important.

All authors designed the study. NK collected the data and wrote the first draft, which was then improved and revised by all authors.

The authors would like to thank Tomasz Czuba and Elisabet Berg for statistical consultations.

*Acta* thanks Tanja Kraus and Terje Terjesen for help with peer review of this study.
